# Chimpanzee utterances refute purported missing links for novel vocalizations and syllabic speech

**DOI:** 10.1038/s41598-024-67005-w

**Published:** 2024-07-25

**Authors:** Axel G. Ekström, Charlotte Gannon, Jens Edlund, Steven Moran, Adriano R. Lameira

**Affiliations:** 1 Speech, Music & Hearing, KTH Royal Institute of Technology, Lindstedtsvägen 24, 118 28 Stockholm, Sweden; 2 Department of Psychology, University of Warwick, Coventry, UK; 3 Institute of Biology, University of Neuchâtel, Neuchâtel, Switzerland; 4 Department of Anthropology, University of Miami, Coral Gables, USA

**Keywords:** Anthropology, Animal behaviour, Human behaviour, Evolution, Psychology

## Abstract

Nonhuman great apes have been claimed to be unable to learn human words due to a lack of the necessary neural circuitry. We recovered original footage of two enculturated chimpanzees uttering the word “mama” and subjected recordings to phonetic analysis. Our analyses demonstrate that chimpanzees are capable of syllabic production, achieving consonant-to-vowel phonetic contrasts via the simultaneous recruitment and coupling of voice, jaw and lips. In an online experiment, human listeners naive to the recordings’ origins reliably perceived chimpanzee utterances as syllabic utterances, primarily as “ma-ma”, among foil syllables. Our findings demonstrate that in the absence of direct data-driven examination, great ape vocal production capacities have been underestimated. Chimpanzees possess the neural building blocks necessary for speech.

## Introduction

The ability to learn new vocalizations—known as vocal learning—is often assumed to have paved the way for spoken language in human evolution^1,2^. It has long been claimed, however, that nonhuman primates are capable of vocal usage learning (producing pre-existing calls in new contexts), but not vocal production learning (modifying pre-existing signals, socially learning or imitating calls from other individuals)^3–8^. This conclusion has not, however, been reached directly^8^, but instead through second-hand accounts of classic ape-language projects, which explicitly state that subjects *did* learn human words, such as “cup” and “mama”^9–14^ and that some great ape species are more “conversational” than others^15^. Challenging the reputation of great apes as unsuitable models for speech and language evolution, chimpanzees in the wild do not produce any “cup”-like or “mama”-like utterances, suggesting they are indeed vocal learners. In the absence of direct analyses of original recordings, the interpretation that “despite repeated attempts, no nonhuman primates have ever been trained to produce speech sounds, not even chimpanzees raised from birth in human homes”^16^ has paradoxically become a prevailing belief. It has also led to extrapolations that great apes lack key neural circuitry for voluntary motor control over the voice and articulators (i.e., lip, tongue, jaws), as forwarded by the “Kuypers-Jürgens hypothesis”^8,17^. Consequently, vocal production learning has been widely assumed to have emerged anew in the human lineage after it diverged from extant non-human great apes.

Voiced labial articulations, such as “mama”, are among the first words to emerge in human infants during canonical babbling – one of the earliest stages of speech and language development in children^18,19^. The “frame/content” theory of speech evolution^20^ posits that such syllabic cycling originated in mandibular oscillatory behavior employed by extant non-human primates for rhythmic facial gestures such as lip smacking^21^. Both voicing and jaw oscillatory motions are present in most mammals from birth, but human speakers make unique use of these capacities in the ready production of voluntary and combinatorial syllabic speech, where “syllable” is defined phenomenologically, referring to a combination of consonantal “frame” and vowel-like “content”^20^. Were such learned syllabic coupling to be demonstrated in a non-human great ape, it would set back origins of these abilities to an earlier stage of evolution. Here, we show, by way of phonetic analyses and listener experiments, that two chimpanzees—Johnny and Renata—possessed the necessary control of the articulatory organs to produce phonatory-mandibular coupled disyllabic utterances, corresponding to the lexical form “mama”. The chimpanzee Johnny produced four utterances of “mama” (and two seemingly interrupted utterances of “ma”)^22^. Out of the total of six utterances, one of each of “mama” and “ma” were deemed unusable due to excessive interfering noise distorting spectrograms, resulting in a total of three utterances of /mama/ and one utterance of /ma/ being selected for analysis (*N* = 4). The chimpanzee Renata produced four utterances of /mama/ (*N* = 4)^23^.

In speech production, a voice “source” from the vocal folds of the larynx is “filtered” in the supralaryngeal vocal tract, where the movement of articulators (e.g., lips, jaw, and tongue) affect the resonances (termed formants) of the tract—widely recognized as a key factor in the recognition of speech signals by human listeners^24^. Voiced bilabial nasal /m/ (the consonant in “ma”) is accomplished via the occlusion of egressive (outward) airflow in the vocal tract, redirecting it to the nasal cavities. It is voiced, meaning that vocal cords vibrate actively during production (phonation), and it is articulated bi-labially (using both lips). Notably, /m/ forces airflow to a sudden near-stop, with resonances reverting closer to the frequency of infinite impedance (i.e., zero air flow) of the oral cavity behind the lips^24^. These phenomena are readily observable in sound spectrograms because the relationship between speech articulation and speech acoustics is non-monotonic^25^; for some regions in articulatory space, the resulting acoustic signal remains relatively stable as articulatory variables change. For others, slight changes in articulation result in abrupt acoustic changes: the transition from closed mouth to open mouth in /m/ represents such a sudden change. In comparison, in /w/ (“wah”), formants display a tell-tale transitional glide as the mouth opening narrows and widens without reaching complete closure. While chimpanzees in the wild produce lip smacks^26^, there are no indications of /m/-like (a voiced bilabial nasal utterance) or /ma/-like utterances in the chimpanzee vocal repertoire^27,28^. Verified utterances of /mama/ by chimpanzees would thus be a case of vocal production learning^3–8^.

## Methods

### Who were Johnny and Renata?

Johnny the chimpanzee featured in a home video recorded whilst living with the Suncoast Primate Sanctuary at Palm Harbor, FL, US. The footage is publicly available and, at the time of accessing, had been viewed 447,490 times. To our knowledge, it represents the only available recording of Johnny’s utterances. According to the video information, Johnny passed away in 2007. Johnny’s “mama” utterances are seemingly prompted by the woman recording the video asking, “Can you say mama?”, implying (though not definitively) that these utterances may have been initially learned through imitation. In the video comment section, the owner of the account posted, “Johnny called everyone Mama”, and claimed that Johnny “knew that [saying] Mama would get him anything he wanted as long as it was on his diet…” We may infer that Johnny’s “mama” utterances, whatever their origin, appear to have been sustained through reinforcement (i.e., rewards for given behavior). Renata the chimpanzee was featured in the film “Now Hear This! Italians Unveil Talking Chimp”, released in 1962 as part of Universal Studios’ *Universal Newsreel* series of newsreels. The footage is publicly available and, at the time of access, had been viewed 97 times. The ultimate fate of Renata is not known to us. Similarly to Johnny’s utterances, in the relevant segment, Renata’s handler is seen tapping Renata’s chin as an apparent behavioral cue, also consistent with reinforcement learning. We are not aware of any context as to how these utterances were learned. Both recordings were downloaded from Youtube in .wav format, and not otherwise pre-processed prior to analysis. While we analyzed utterances by captive animals, our data were sourced from archival footage. As such, our work is in accordance with all relevant institutional guidelines.

### Listening experiment

A listening experiment was programmed in the online platform Qualtrics XM Platform with the aim of assessing human perception of utterances. Ethical approval was obtained prior to data collection, informed consent was obtained prior to participation in the online perception experiment and the participants were adequately debriefed after the experiment. This data collection procedure was approved by the University of Warwick Department of Psychology Research Ethics Committee.

Chimpanzee utterances (*N* = 2, 1 from each of Johnny and Renata) were mixed in with Spanish-language Parkinsonian speech utterances^29^. Participants were instructed that the utterances were from human speakers diagnosed with speech pathologies. Parkinsonian speech is characterized by delayed and imprecise articulation and dysphonic phonation, compared to healthy controls^29,30^. The purpose of the presentation scheme was not to prompt the listeners’ perception of the chimpanzee utterances as speech-like—human perception is sensitive enough to perceive even non-speech sounds as phonemic^31^—but to mask their otherwise “inhuman” quality. All Parkinsonian utterances were disyllabic, matching the chimpanzee utterances for apparent syllable count. Because the chimpanzee utterances were contextually noisy, we masked each Parkinsonian utterance using “speech-shaped” noise^32^ in *Audacity* (audacityteam.org). Such “masking” procedures are commonplace in research on speech perception^33,34^ and speech intelligibility^32^.

In the listening experiment, each utterance was presented in isolation, and participants had the opportunity to freely replay each one at their own discretion. Participants were asked to provide orthographic transcription in letters (i.e., “mama”, “mawa”) for each utterance. If participants perceived and transcribed the chimpanzee utterances similarly to “ma” or “wa”, respectively, it would support our phonetic analyses of the chimpanzees’ utterances as essentially corresponding to human words. On the other hand, if the coding of the chimpanzee utterances contradicted our phonetic analyses (or if ratings were simply inconsistent), it would imply they were too contextually noisy to reliably transmit linguistic information.

### Coding procedure and exclusion criteria

Transcriptions that indicated di-syllabic utterances (“mama”, “nya-nya”) were treated as valid data. For example, “mama”, “ma-ma” and “mamma”, were all transcribed as /mVmV/. We applied the same criteria and procedure while coding transcriptions of chimpanzee and human utterances.

In languages that use the Latin alphabet, consonants “m”, “n”, “p”, and “b” typically correspond phonologically to /m n p b/, with comparatively minor differences. Assessing agreement between vowel transcription offers unique challenges. Lack of agreement over vowel transcription does not necessarily imply disagreement per se. Relevant research^27,35^ indicates that chimpanzees often make use of a mostly “open” vocal tract, resulting in the articulatory equivalent of unstressed vowel schwa /ə/. For our subjects, while we do not have access to reliable measurements of the animals’ size, we may make rudimentary estimates. For example, for one of Renata’s utterances (R_4mama38461.wav), we estimated the first spectral peak (or formant) at approximately 800 Hz and the second at approximately 1900 Hz, roughly corresponding to /æ/ (the vowel in “cat”) spoken by an adult male speaker. The presence of a human in the film lets us infer that Renata’s stature appears rather small, however, and that she may not be a fully grown individual.

A number of works are concerned with the capacities of primates to articulate vowels^16,36–38^, and it has been known for decades that “the chimpanzee vocal tract [has] the anatomic ability to … produce a number of vowels that in human speech are ‘phonemic elements’ (^36^, pg. 299). However, underlying biomechanics governing the realistic production of any such vocalizations are uncertain^39,40^. Accordingly, we may no more than speculate on the articulatory configurations employed by our subjects. However, we note that the vowel-like signals in Renata’s utterances seemingly correspond to a short rather open vocal tract with a “flared” oral cavity^35^ and possibly a tongue retracted to narrow the anterior oral cavity, shifting up the second resonant frequency^24^.

With regards to transcription, however, vowel phonemes in close-to-mid central region of the vowel space are far from uniformly represented by the same symbol across languages (^41^, pg. 95–96). While languages may use the same letter in written language (e.g., “a”), they may be realized disparately both within and between languages in real-life speech. In addition, our sample was diverse with regard to listeners’ native languages. Indeed, even within languages, the correspondence between written symbols and uttered sounds is highly inconsistent. In English alone, the symbol “a” may correspond to a range of different sounds, including the /æ/ in “c**a**t”, the /eɪ/ in “block**a**de”, /a:/ in “f**a**ther”, or schwa /ə/ in “**a**bout”. However, letters “a”, “u” (“s**u**pply”), and “o” (“el**o**quence”) more commonly correspond to schwa, compared to e.g., “i” (though there are examples of “i” corresponding to schwa, for example, the “i” in “penc**i**l”). Accordingly, a higher proportion of symbols “a”, “o”, and “u” versus “i” or “y” may reasonably be taken as indicative of agreement in a broader sense. However, in order not to artificially inflate indications of agreement, for vowels, we assess agreement by the three most transcribed letter(s) for each syllable.

Our coding procedure, thus, was as follows:

1. Code all transcriptions as indicative of perceived place and manner of articulation; for example, “mama” will be coded as /mama/.
2. Code diphthongs (two-vowel syllables, such as “ai” /ai/) as such, and not per its individual components (i.e., /a/ and /i/ separately).
3. Similarly, where a syllable is transcribed as composed of two consonants—for example, “fnaya”—code both consonants: in the example case, the first syllable consonants are to be coded as /fn/, rather than /f/ or /n/.
4. Where transcriptions run counter to instructions, and indicate real words—for example, “my house”—code these data as n/a. Similarly, where transcriptions indicate three- or four-syllable words, code these data as n/a; revisit these data, to determine the cause of these perceptions.
5. Compute the percentage of agreement between participants.

## Results

### Phonetic analyses

**Figure 1:**
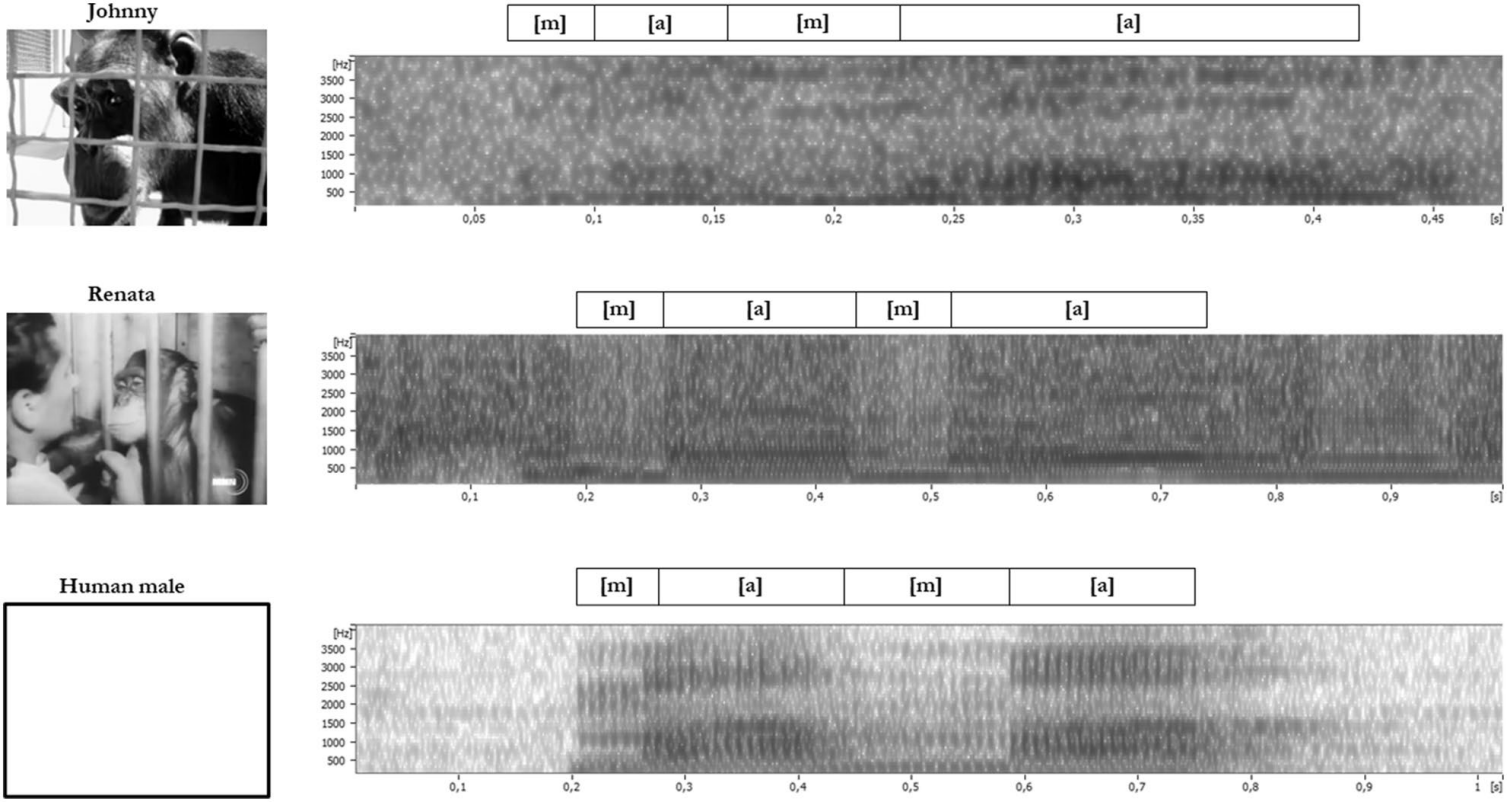
Spectrograms of utterances by Johnny (top), Renata (middle), and /mama/, uttered by one of the authors (bottom). Sampling frequency of all files was 44,1 kHz. Frequency range 0–4 kHz. Window length 5 ms. Time scales 0–500 ms (top), 0–1000 ms (middle), and 0–1010 ms (bottom). Johnny’s utterances appear with formant transitions indicating incomplete lip closure. Renata’s utterance exhibits rapid formant transitions, consistent with /mama/, as uttered by one of the authors. Utterances by both chimpanzees show voicing across the utterances, consistent with consonant–vowel–consonant–vowel cycles, implying consistent and overlapping innervation of the larynx and jaw. Spectrograms rendered in *Audacity.*

Johnny appeared to produce [m] as the word-initial syllable “mama”, most clearly indicated in the singular utterance of “ma”, though for several utterances, the spectrographic consonantal profile of the word-initial syllable is more consistent with voiced labial-velar approximant /w/, indicating incomplete or inconsistent lip closure. Thus, Johnny was seemingly alternately producing “wama” or “mama”. These features were readily identifiable using methods designed for analysis of human speech, with Johnny unequivocally employing simultaneous phonation (i.e., voicing) and articulation using the jaw and lips to produce /ma/ and /wa/^26,42^. Like Johnny, Renata also demonstrates coupling of phonation and articulation. However, Renata’s utterances consistently display the sudden stop and redirection of acoustic energy consistent with complete bilabial closure (“ama”), for both word-initial and word-final would-be syllables (Fig. 1). Renata reliably produced “mama”.

### Listening experiment sample characteristics

Our sample consisted of 33 women and 28 men (*N* = 61), aged between 18 and 71 (*M* = 34.67, *SD* = 13.24) and was gathered through convenience sampling. The most represented groups were native speakers of English (~ 32.79%), Swedish (~ 22.95%), Dutch (~ 9.83%), Spanish (~ 9.83%), and Italian (~ 4.92%), though the sample also included native speakers of German, French, Portuguese, Russian, Hungarian, Gujarati and Arabic. In addition, to control for possible training effects of significant exposure to phonetics, participants were also asked to state whether they had any previous experience with phonetic transcription. Of these, 8 people (13.11%) reported “extensive” experience, 22 reported “some” experience (36.07%), and 31 (50.82%) reported “none”, suggesting a largely even split between trained and untrained participants.

### Human listeners’ transcription

Study participants largely agreed as to transcriptions of consonant phonemes for the chimpanzee utterances, reaching agreement around ~ 75% that the sound corresponded to /m/ (Tables 1, 2). There was more disagreement for Johnny’s utterances (which were seemingly executed with incomplete mouth closure). The second and third most transcribed consonants for the first syllable in Johnny’s utterance, was a *vowel* phoneme (~ 13.11%) or voiceless glottal fricative /h/ (~ 8.2%) (the first consonant in “**h**igh”). This is reasonably consistent with our phonetic analysis where we note that Johnny’s utterance is seemingly produced with incomplete lip closure, more closely corresponding to /w/ (“**w**ah”). /n/ was a relatively common substitute (~ 4.92%). Other labial consonants /p/ (“**p**ah”), /b/ “**b**ah”) were also observed in this position. There is no alternative set of inclusion criteria that results in counter to those presented (Table 3).

**Table 1:** Most common consonants and vowels transcribed for Johnny’s and Renata’s utterances.

	1st C	1st V	2nd C	2nd V
Johnny				
Phoneme	/m/	/a/, /o/, /u/	/m/	/a/, /o/
%	57.1%	60.7%, 19.6%, 14.3%	85.7%	91.1%, 8.9%
Renata				
Phoneme	/m/	/a/, /e/, /o/	/m/	/o/, /a/, /au/
%	80.6%	64.1%, 12.8%, 7.7%	75%	25.6%, 20.5%, 20.5%

**Table 2:** Inferred place of articulation for would-be consonants in chimpanzee “mama” utterances. /m w p b/ are labial sounds. Results show that would-be consonants are generally perceived as produced with labial or bilabial place of articulation.

	Johnny’s “mama”				Renata’s “mama”			
	1st consonant		2nd consonant		1st consonant		2nd consonant	
	Count	%	Count	%	Count	%	Count	%
/m/	32/61	52.5%	48/61	78.69%	29/61	47.5%	27/61	44.3%
/n/	3/61	4.92%	4/61	6.56%	5/61	8.2%	6/61	9.84%
/w/	1/61	1.64%	2/61	3.28%	0/61	0%	0/61	0%
/p/	1/61	1.64%	0/61	0%	0/61	0%	0/61	0%
/b/	1/61	1.64%	0/61	0%	0/61	0%	0/61	0%
/h/	5/61	8.2%	1/61	1.64%	0/61	0%	0/61	0%
/f/	0/61	0%	0/61	0%	1/61	1.64%	0/61	0%
/d/	0/61	0%	0/61	0%	0/61	0%	1/61	1.64%
/r/	0/61	0%	0/61	0%	0/61	0%	2/61	3.28%
V	11/61	18%	0/61	0%	0/61	0%	0/61	0%
Other	2/61	3.28%	1/61	1.64%	1/61	1.64%	0/61	0%
N/a	5/61	8.2%	5/61	8.2%	25/61	41%	25/61	41%

**Table 3:** Most common consonants and vowels transcribed for Johnny’s utterances, Renata’s utterances, with and without including n/a values.

	1st C	1st V	2nd C	2nd V
Johnny				
With n/a’s				
Phoneme	/m/^a^	/a/, /o/, /u/^a^	/m/^a^	/a/, /o/^a^
%	52.5%	55.7%, 18%, 13.1%	78.7%	83.6%, 8.2%
		86.8%		91.8
Without n/a’s				
Phoneme	/m/^b^	/a/, /o/, /u/^b^	/m/^b^	/a/, /o/^b^
%	57.1%	60.7%, 19.6%, 14.3%	85.7%	91.1%, 8.9%
		94.6%		100%
Renata				
With n/a’s				
Phoneme	/m/^a^	/a/, /e/, /o/^a^	/m/^a^	/o/, /a/, /au/^a^
%	47.5%	41%, 8.2%, 4.9%	44.3%	16.4%, 13.1%, 13.1%
		54.1%		42.6%
Without n/a’s				
Phoneme	/m/^c^	/a/, /e/, /o/^d^	/m/^c^	/o/, /a/, /au/^d^
%	80.6%	64.1%, 12.8%, 7.7%	75%	25.6%, 20.5%, 20.5%
		84.6%		66.6%
After relabeling with n/a’s				
Phoneme	/m/^a^	/a/, /e/, /o/^a^	/m/^a^	/au/, /u/, /o/^a^
%	73.8%	67.2%, 8.2%, 6.6%	67.2%	18%, 16.4%, 16.4%
		82%		50.8%
After relabeling without n/a’s				
Phoneme	/m/^e^	/a/, /e/, /o/^b^	/m/^b^	/au/, /u/, /o/^f^
%	81.8%	73.2%, 8.9%, 7.1%	73.2%	19.3%, 17.5%, 17.5%
		89.2%		54.3%

^a^*N* = 61, ^b^*N* = 56, ^c^*N* = 36, ^d^*N* = 39, ^e^*N* = 55, ^f^*N* = 57

In contrast to patterns of transcription observed for Johnny’s utterances, Renata’s word-initial syllable “ma-” was never transcribed as vowels or /h/. This, too, is consistent with our phonetic analyses and the conclusion that Renata’s “m”s were consistent with complete labial closure, as evident from abrupt redirection of energy between “m” and “a”. For Renata’s utterances, upon analysis, a large subset (~ 41%) of data were labeled as n/a. These data were coded as such because they were written as three- or four-syllable “words.” Upon inspection, we observed that for many three-syllable or four-syllable transcriptions, the utterances were transcribed as a sequence of syllables, where a seemingly random burst of noise later transitioned into an ostensibly “mama-like” form—for example, “kuma-mao”, “Ash-ma-ma”, or “Homo-mo”. This indicates that noise prior to the utterance proper had been interpreted by a minority of listeners as *part* of that utterance. To assess this possibility, we introduced an additional coding criterion:

1. In three- or four-syllable transcriptions, the two transcription-final apparent syllables were coded as in^1^; for example, “Homo-mo” was coded as /momo/.

This relabeling resulted in a significantly lower percentage of n/a data (~ 8.45%, averaged across all four phonemic positions). However, because this also introduced a risk of artificially inflating our data, we calculated percentages both including and excluding n/a data (same procedure as for Johnny’s utterances, described above), and as well as this additional “liberal” interpretation (i.e., treating the two final syllables of a subset of n/a data, as permissible) (Table 2).

For transcribed vowels, there was less apparent agreement. This likely reflects the international nature and broad linguistic background of our sample. Analysis of ratings suggests that both Johnny and Renata likely produced versions of “unstressed” schwa /ə/^25,35^ (though the frequency of the second formant in Renata’s utterances may also be suggestive of a slightly retracted tongue). Our data show higher proportions of symbols “a”, “o”, and “u” versus “i” or “y”, which is consistent with cross-linguistic transcription for schwa^41^.

In summary, we took several precautions to avoid inflating agreement in our listener data. Regardless of the coding scheme applied, data consistently provide support for our interpretation. For all chimpanzee would-be consonants, “m” was the most consistently transcribed interpretation. Transcription for vowels was more variable, possibly reflecting the diverse linguistic background of our sample. Our data, while variable with regard to at least one of the vowels (Renata’s word-final syllable), was typically transcribed as “au”, “u”, or “o”, rather than—for example—/i/ (“s**ee**”) or /y/ (**ü**ber) (Table 2). Finally, the data analyzed in this study were “found data”—we did not have any input on the circumstances of their recordings or the animals’ behavior. Thus, recording quality may have served as a depressor of agreement between listeners. Given control over recording conditions and direct contact with the animals, our listener data would likely show greater agreement.

## Discussion

### Expediting the gift of gab

Our results add to the emerging discussion on the evolution of the “vocal brain”^43,44^. In particular, findings reported here suggest that aspects of the neurological audiovocal system—the study of which has often assumed convergent evolution in human and songbird lineages—may have much older origins than previously thought^3–8,44^. Our results falsify two facets downstream of the “Kuypers-Jürgens hypothesis”^8,17^—the theory positing that a lack of control of the vocal apparatus precludes vocal learning. First, and most evidently, our data are evidence of learned novel vocalizations by chimpanzees. That is not to claim that there have been no neurological changes in the human brain that facilitate speech production, however; one meaningful example is the “progressive increase in size and complexity”, from chimpanzees to humans, of temporo-frontal connectivity (e.g., the arcuate fasciculus) associated with a capacity for vocal imitation^44,45^. However, our findings caution that whatever changes may be observed in ape-human neurology does not allow for unreservedly inferring an evolutionary timeline toward speech without dedicated research effort and direct evidence from great ape vocal behavior.

Second, Brown and colleagues (^46^*, p.* 1020) speculate that overlap between somatotopic cortical representations of larynx and jaw represented “the critical evolutionary step to develop syllable structure from a precursor of mandibular oscillations … creating an evolutionary transition from [primate] lip smacking to something like the ba-ba-ba sound of human babbling by means of voice/jaw coupling.” Our data definitely demonstrate that chimpanzees have passed this “critical evolutionary step”: while undoubtedly a crucial underpinning of speech production, the hypothesized missing link precluding chimpanzees from voluntary jaw-voice coupling evidently does not exist.

Our recovered chimpanzee recordings involved two unrelated individuals of different sexes, living in different time periods, on different continents, but producing the same lexical form: “mama”. These two cases clearly align with ape language projects that repeatedly reported “mama” as one of the words vocally learned by ape subjects^9^ but dismissed in the absence of rigorous analysis^3–8,16^. The phoneme /m/ is ubiquitous in human languages^47,48^ and is among the first speech sounds to be produced in human ontogeny, sometimes as early as two months of age^18^. This early-in-life occurrence results in part from infant vocal anatomy limiting possible articulations^19,49–51^, making /mVmV/ (m–vowel–m–vowel) cycles among the first available multisyllabic utterances in an infant’s repertoire. Low front vowels are among the first to be produced by developing human infants^19^ and require little deliberate independent recruitment of lingual musculature. Repeated iterations of single-syllable sequences such as “mamama” occur in human infants as part of the canonical babbling stage and is replaced by sequences of contrasting syllables in the variegated babbling stage towards the end of the first year of life. Accordingly, it has been argued that “mama” may have been among the first words to appear in human speech^20,52^. Our data complements this picture: chimpanzees can produce the putative “first words” of spoken languages.

### Chimpanzees outperform other mammals—by sounding more human

The lexical form “mama”, when spoken by chimpanzees, exhibits phonetic features typical of the same utterance when produced by human speakers, and are perceived as contextually appropriate syllables by human listeners. These results corroborate a growing body of evidence that great apes are vocal production learners^8,14,53–55^, dispelling decades-old misconceptions about the species’ voice and articulatory control, and by extension, their value as comparative models for speech and language evolution^3–8^. Because ours are secondary data, sourced from historical footage and not collected in circumstances of experimental control, we may not ascertain how these two chimpanzee subjects acquired their novel speech-like vocalizations. We may, however, draw important comparisons.

Literature on vocal learning has so far been concentrated on cases reported for distantly related species, such as elephants^56^, beluga whales^57^, and mynah birds^58^. However, these are cases of vocal *emulation*. These species do not produce speech-like utterances in ways that mirror those of human speakers, but rather achieve comparable acoustic outcomes by employing highly disparate articulatory maneuvers. Our data, meanwhile, showcases apparent vocal learning employing anatomically homologous vocal morphological structures. Stoeger et al. summarize that, for listener agreement over speech-like utterances by an elephant, “agreement was high for vowels [at 67%] and relatively poor for consonants [at 21%]”^56^. In our study, listeners agreed to a greater extent regarding consonants, at ~ 71.4% for Johnny; and ~ 77.8% for Renata (Table 1). Because chimpanzees share much of the relevant articulatory morphology with humans—large “fleshy” lips, subject to independent and voluntary control (^14,26–28,42,59^—the acoustic effects of lip and jaw movement are highly similar between humans and chimpanzees when uttering comparable phonetic forms. If reproducing human words or phonetic contrasts is a qualifier for vocal learner status, and if the modest success of an elephant meets that criterion^56^, we must extend the same distinction to chimpanzees, who are capable of producing phonetic contrasts at higher levels of human perceivability.

### Revisiting “ape language”

Great ape language projects have been misrepresented in the literature. The few ape subjects involved have mistakenly been depicted as trustworthy representations of the capacities of their entire genus. Our findings show that interpretation of these classic studies must be done with caution. Namely, absence of evidence (i.e., what these individual animals were purportedly incapable of doing) should not be taken as evidence of absence. Fifty years after these projects, caretakers who looked after the welfare of the great apes during these projects are documenting and re-examining the “neglect and cruelty inflicted on [these] animals on the quest of psychological study”^60^. Subjects in “ape language” studies were traumatized, their emotional, ecological and social needs unmet, with many “being captured in the wild [after the murder of their mother], subjected to unhealthy and unnatural environments and starved of modeling of healthy group behaviors”^60^. Current discussions on the evolution of speech and language have nevertheless continued to base their assumptions on these studies^3–8^ while disregarding a new generation of ethically approved studies conducted in accredited animal-welfare institutions and in the wild^8,14,47,48,61^. Great apes can produce human words; the failure to demonstrate this half a century ago was the fault of the researchers, not the animals.

## Data Availability

All data and materials used in this study are publicly available in the relevant GitHub depository <github.com/evofant/ChimpanzeeMissingLink>.
